# Simulation and optimization of the thermal sterilization process of puree cans using the production of chestnut puree as an example

**DOI:** 10.3389/fmicb.2023.1135700

**Published:** 2023-04-24

**Authors:** Wencheng Wang, Jinqing Wu, Jiali Zheng, Zhiliang Wu, Jinfeng Huang, Yibin Lu, Xiaoyan Peng, Liqing Huang

**Affiliations:** ^1^Zhangzhou Institute of Technology, Zhangzhou, Fujian Province, China; ^2^Zhangzhou Food Industry Research Institute, Zhangzhou, Fujian Province, China; ^3^Fujian Zishan Group Co., Ltd., Zhangzhou, Fujian Province, China

**Keywords:** thermal sterilization, *F* value, COMSOL, numerical simulation, model

## Abstract

In the production process of puree cans such as chestnuts cans, it is easy to browning due to excessive heating, which causes a lot of waste every year. The heat and mass transfer model of Chinese Chestnut Puree was established through the finite element method. The model simulated the change process of the temperature field, heat flow velocity field and *F* value during the production of Chinese Chestnut Puree. After comparing and confirming the effectiveness of the model through the thermal penetration test, the model was used to adjust and optimize the production process. For #9121 cans, the two-stage sterilization method was adopted. Through the sterilization method at 10–65–48-14/118–110°C, a sterilization effect equivalent to that of the original process at 10–86-24/121°C was achieved, the browning problem of the product was alleviated, and the product quality was improved. This practice can also provide a reference for canning enterprises to adjust their production processes in the future.

## 1. Introduction

Puree cans are canned products made by crushing and stirring food into pulp and placed through certain thermal sterilization procedures. Such products are often used as semifinished products in cold drinks, desserts, baking products and other foods, which are deeply loved by consumers and have very large market sales. However, due to the high viscosity and poor fluidity of most puree cans, the color will darken near the edge of the can wall after being made into cans for sterilization, especially for products with lighter colors, such as taro puree and chestnut puree. Enterprises that use this kind of product in the later stage usually deduct and discard this part of the products, which causes a large amount of waste every year.

## 2. Background and scope

This phenomenon may be caused by the unreasonable sterilization process, which leads to excessive edge temperatures and then leads to intense Maillard, caramelization, and other nonenzymatic reactions (Martins et al., 2000; Corzo-Martínez et al., 2012; Nooshkam et al., 2019; Sen and Gokmen, 2022). Therefore, to study this issue, it is necessary to formulate a more reasonable sterilization procedure for cans (Kannan and Sandaka, 2008; Simpson, 2009; Hayakawa, 2010). However, canning sterilization technology is very critical for controlling the safety of canned products (Goldblith, 1971). On the one hand, a suitable sterilization process requires the production of commercial sterile products; on the other hand, it requires that the products have better edible value and nutritional value (Ball and Olson, 1957; Manson et al., 1970; Stumbo, 1973; Ramesh et al., 1997a; Wal, 2003). To evaluate the degree of sterilization of cans through heating, we mainly evaluated the relationship between the sterilization temperature, sterilization time and the lethal rate of the target bacteria (Ramesh et al., 1997b; Chen et al., 2005). Moreover, we used the general method or classical method to calculate the sterilization conditions. This method utilizes the lethal rate of the target microorganism in the whole process of thermal sterilization; furthermore, this method evaluates its final sterilization effect with its value to calculate the reasonable combination of sterilization temperature and time conditions and determine the specific sterilization condition parameters through validation tests (Teixeira, 1969; Corradini et al., 2005). In terms of the sterilization prediction of cans, the ball formula method (Ball and Olson, 1957), the Stumbo formula method (Stumbo, 1973), and the Hayakawa formula method can be used (Hayakawa, 1977). These methods have been adopted to a certain extent, but due to their long history, the early stage relies on experience, mostly through summary and induction (Holdsworth and Simpson, 2016; see Table 1).

**Table 1:** Nomenclature used in this study.

Nomenclature			
C_P_	specific heat of the liquid in the can J/(kg K)	APA	After production process adjustment
C_nc_	Natural convection constant	BPA	Before production process adjustment
E_a_	Activation energy (kJ/kg·mol)	t	time of heating (s)
f	Force (N)	T	temperature (K)
F	*F* value (min)	T_0_	initial temperature inside food can (K)
F_o_	Fourier number	T_wall_	temperature at the wall of the can (K)
g	Gravitational acceleration constant (m·s^−2^)	T_i_	initial volume-averaged temperature (K)
H	Height of the food can (m)	P	Pressure (N)
h	Heat transfer coefficient (W/m^2^ K)	Pr	Prandtl number
k	Thermal conductivity of the liquid in the can (W/m K)	q	Conduction heat flux (W/m^2^)
n	Exponent to shear rate in dynamic viscosity expression	Q	heat source (W/m^3^)
Nu	Nusselt number	u	Heat flux velocity vector (m/s)
NOZ	Non overheated zone	R	radius of the food can (m)
OZ	Overheated zone	Ra	Rayleigh number
SHZ	Slow heating zone	Z	Z value (10°C)
TZ	Top zone	ρ	Density (kg/m^3^)
		μ	apparent viscosity (Pa s)

In recent years, with the development and application of fluid computing software, many scholars have tried to guide production practices through computer simulation (Singh et al., 2015, 2017). For example, by using computational fluid dynamics (CFD) software to simulate changing the geometry and orientation of the container, the temperature of the slowest heating zone (SHZ) of three geometries was tracked and compared, and then the method of enhancing natural convection heat transfer in the sterilization process of canned food was studied (Varma and Kannan, 2005, 2006). Taking canned pineapple as the research object, the influence of product size reduction on the heat transfer effect during heat treatment was studied (Padmavati and Anandharamakrishnan, 2013). The flow pattern of brine and the temperature evolution of olive oil in brine during heating and cooling were calculated through the CFD program, and the distribution of the temperature and *F* value in several cases, as well as the location of the slowest heating zone and critical point in the product, were evaluated (Dimou et al., 2013). The effects of the particle orientation on the fluid flow, temperature evolution and microbial destruction during the hot processing of semi-canned syrup peaches were studied (Dimou et al., 2014). Using the ANSYS fluent computational fluid dynamics program, the three-dimensional pasteurization simulation of canned apple puree was carried out at 378 K in a semirigid aluminum-based container. The influence of the grid structure on the temperature distribution during heat treatment and the influence of the headspace (steam) on heat transfer were studied (Shafiekhani et al., 2016). Through COMSOL Multiphysics software, taking water and Carboxymethylcellulose solutions as the research objects, the temperature distribution, velocity distribution and lethal rate value of the whole can space were simulated (Wang et al., 2015). The thermal sterilization of tuna cans was numerically simulated, two models of pure conduction and solid–liquid mixing were established, and the differences between them were compared (Liang et al., 2015). The results showed that the pure conduction model significantly underestimated the temperature transfer in the can, and the prediction results of the solid–liquid mixing model were very consistent with the experimental data.

Taking the production of chestnut puree as an example, the two-dimensional axisymmetric model and the finite element method are used in this paper to study the internal heat and mass transfer process of clay cans, predict the sterilization mortality of cans, analyze the causes of can wall browning, and then adjust the sterilization process to provide a reference for enterprises to develop similar puree can products.

## 3. Details of systems and can geometry

### 3.1. Can model

The tin can ^#^9121 is selected as the model to simulate the sterilization process of Chinese chestnuts. The inner diameter of the can is 98.9 mm, the height is 121 mm, and the wall thickness is 0.2 mm.

According to the characteristics of can storage processing and the current production sterilization formula of enterprises, the initial temperature is 68°C and 10–86-24/121°C, the modeling is carried out, and the following assumptions are made according to the actual test situation.

1.According to the conventional average top gap range of cans, it is assumed that the top gap is 5 mm, and the top gap is gradually filled with hot and humid water vapor after heating.

2.Assuming that the density of canned chestnut puree is uniform.

3.It is assumed that the temperature field around the can is symmetrical and uniform, and the chestnut puree and hot and humid steam in the can are uniform and symmetrical.

4.The influence of the probe and fixture on heat transfer and heat flow is ignored.

5.It is assumed that the temperature in the initial can is uniform.

### 3.2. Experimental procedure

#### 3.2.1. Production process

Chestnut → cleaning → heating → shelling → crushing → adding water → adding sugar (9% of the total mass) → precooking → degassing → canning → initial temperature adjustment → sterilization → cooling → finished product.

#### 3.2.2. Test temperature verification

**Figure. 1:**
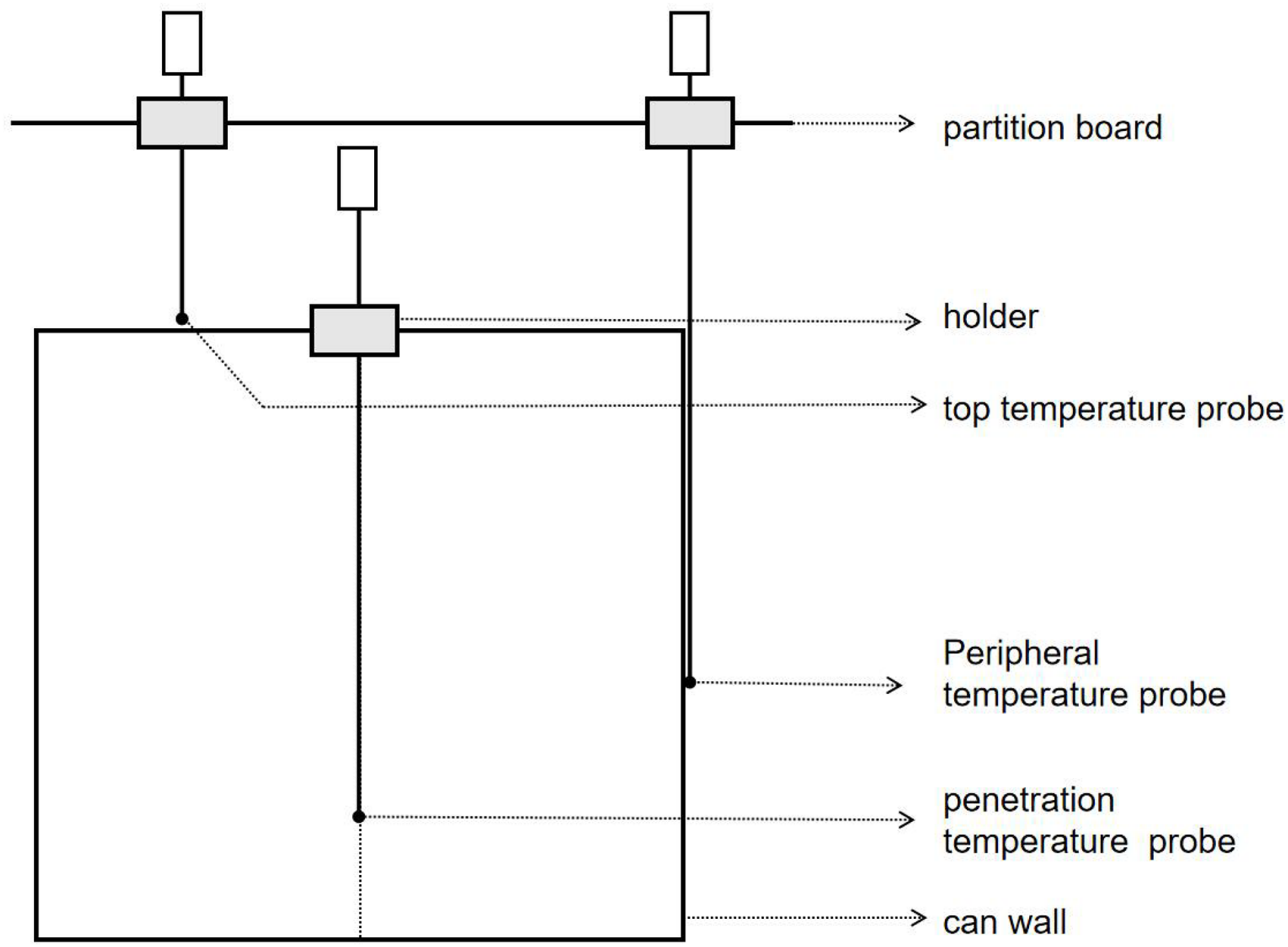
Temperature measuring device.

After determining the corresponding position of the final cold point of cans, a verification test was carried out by the horizontal steam sterilizer. As seen in Figure 1, First, a hole created in the center of the corresponding can cover at the SHZ, the 68°C chestnut puree that is preheated in advance is loaded, the cover is quickly sealed, and the temperature probe is accurately placed 19 mm from the bottom. At this time, the probe temperature is in the range of 67.5 ~ 68.5°C. The metal was placed into a sterilization pot and sterilized at 10–86-24/121°C, and the temperature data were read immediately until the sterilization was finished.

#### 3.2.3. Parameter determination

Canned chestnut Puree has the property of non-Newtonian fluids. The specific heat is determined according to the ASTM E2716-2009, Standard Test Method for Determining Specific Heat Capacity by Sinusoidal Modulated Temperature Differential Scanning Calorimetry (DSC Mettler TGA/DSC3,Switzerland). The dynamic viscosity is determined by referring to ASTM D445-2019 standard (Viscometer, HR/SBQ81834, Beijing HGC Electromechanical Equipment Co., Ltd). The thermal conductivity is measured according to the ASTM D5334-2014 test method for measuring the thermal conductivity of soil and soft rock through hot needle detection (Thermal conductivity tester, DRS-iii, Hunan, China). The density is measured through the pycnometer method. First, the empty bottle (M_1_) was weighed, and then the sample was loaded and weighed again (M_2_). The sample density is the difference between M_1_ and M_2_ divided by the beaker volume. Considering the actual situation, the density beyond the boiling point cannot be measured, and the measurement range is 30–95°C. The density measured at higher temperatures adopts the extrapolation method. The test results of the parameters are shown in Table 2.

**Table 2:** Relevant parameters.

System properties	Value/expression	Unit
Density (ρ)	−0.0000000035 × T^2^–0.4472664761 × T + 1225.5567102981	kg/m^3^
Viscosity (μ)	−0.00000001387024283936 × T^5^ + 0.0000259069861904587 × T^4^ -0.019355480214816 × T^3^ + 7.23213374403098 × T^2^–1351.86899336611 × T + 101200.919774749	Pa·s
Specific heat (C_p_)	3,860	J/(kg·K)
Thermal conductivity (k)	0.982–0.00272 × T + 0.000004531 × T^2^	W/(m·K)

#### 3.2.4. Data processing

Comsol multi-physics 5.6 software was used for model simulation calculation and Auto CAD2022 software was used for volume calculation.

### 3.3. Sensory evaluation

The sensory quality of chestnut paste products before and after the adjustment of the canned sterilization process was evaluated through the method of Shi et al. (2015) and Sidel and Stone (1993), and some modifications were made for reference. Sensory evaluation is based on the aroma, taste, color, tissue state and overall acceptability. Ten well-trained group members were invited and asked to use a 5-part hobby scale. The intensity, preference and overall judgment range from 1 (weak) to 5 (strong), 1 (bad or dislike) to 5 (good or like), and 1 (dislike) to 5 (like). The order of the presentation of samples was random, and scores were expressed as the mean standard deviation.

## 4. Governing equations and numerical solution methodology

### 4.1. Solid heat transfer module

The chestnut puree in the can is affected by the heat transfer of the outer wall, which meets the following requirements:


(1) $$\rho cp\frac{\partial T}{\partial t}+\mathrm{\rho cpu}\cdot \nabla T+\nabla \cdot q=Q$$

where *ρ* indicates the density, kg/m^3^; *C*_p_ Indicates constant pressure hot melting, J/(kg · K); T Indicates temperature, K; *u* Indicates the heat flow velocity vector, m/s; *q* Indicates the conduction heat flux density, W/m^2^; Q represents heat source, W/m^3^.

### 4.2. Fluid heat transfer module

The top gap in the can is filled with hot and humid water vapor after heating, and its steam pressure is a function of temperature. At the beginning of heating, the temperature of the chestnut puree in the can and the can wall is uniform, and the temperature is the initial temperature. According to the Navier–Stokes equation of momentum conservation and the continuity equation of mass conservation (Noguchi, 2019), the following equations are obtained:


(2) $$\frac{\partial \rho u}{\partial t}+\nabla \left(\rho \cdot uu\right)=-\nabla P+\nabla \cdot \left[\mu {\left(\nabla u+\nabla u\right)}^{T}\right]+f+\rho g$$

(3) $$\frac{\partial \rho }{\partial t}+\nabla \cdot \left(\rho u\right)=0$$

where f represents the force on the fluid, *N*; ∇.[*μ*(∇*u* + ∇*u*)^T^] indicates the viscous force, *N*; ∇*P* indicates the pressure, *N*; and *ρg* indicates the gravity of the fluid, *N*.

### 4.3. Dynamic calculation of the *F* value and determination of the cold point position

According to the calculation principle of the enterprise sterilization *F* value, the lethal rate is calculated for the difference between the dynamic temperature value of each point in the process of sterilization and the corresponding microbial lethal temperature, and the time is integrated to solve the dynamic *F* value. Then, by looking for the method of domain minimum value, the SHZ of the can is obtained through this method (Simpson et al., 2015; Rodríguez-Ramos et al., 2021).


(4) $$F=\overset{t}{\underset{0}{\int }}10^{\frac{T-RT}{Z}}dt$$

where F indicates the lethal rate, min; Z value is taken as 100C with Botox as reference; T indicates the temperature somewhere in the can, 0C; RT refers to the reference temperature, 121.10C.

## 5. Analysis of the results

### 5.1. Model prediction and analysis

#### 5.1.1. Temperature prediction and analysis during sterilization

**Figure. 2:**
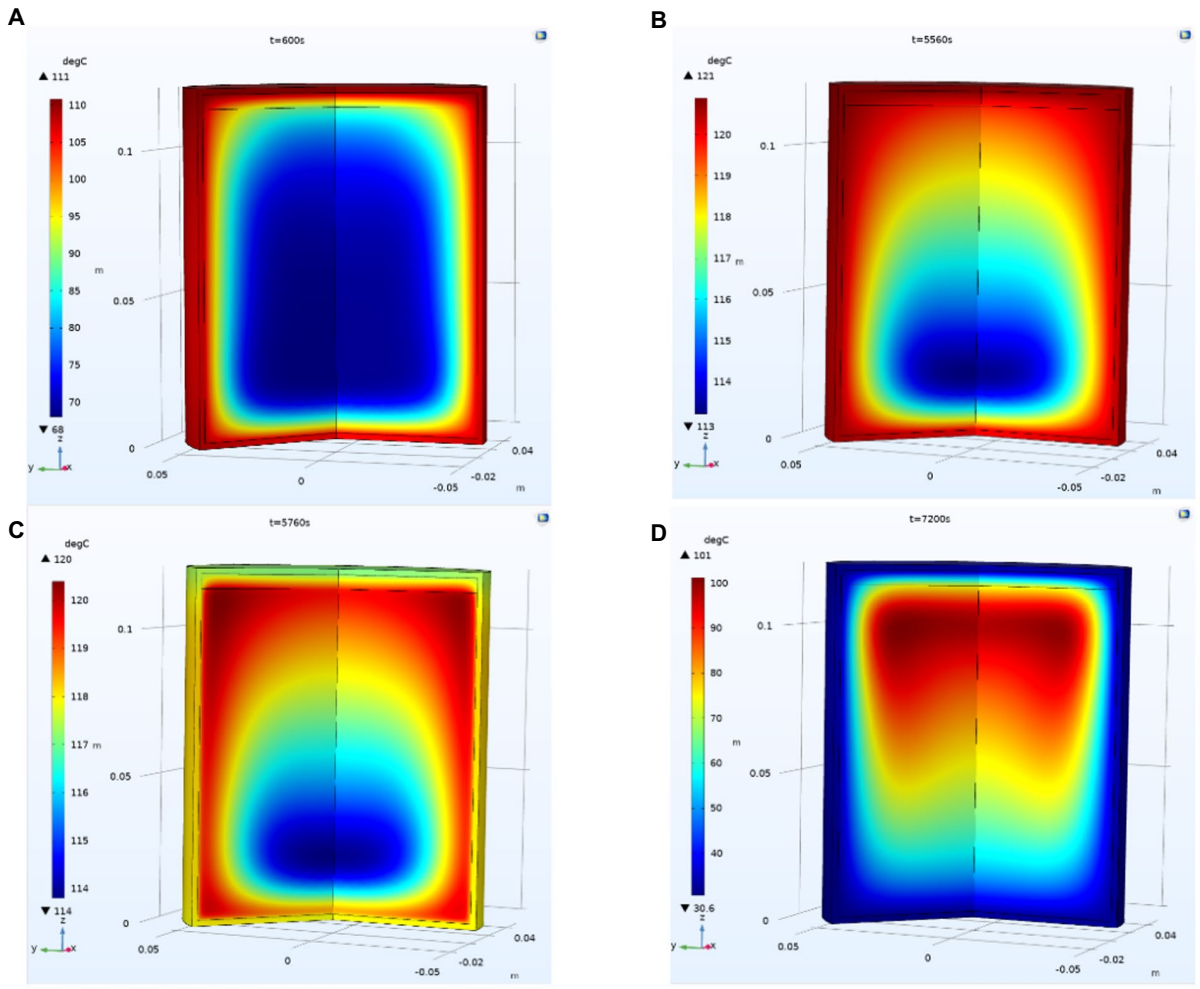
Change in the temperature field in the can. **(A)** Temperature distribution in can when *t* = 600 s; **(B)** Temperature distribution in can when *t* = 5,560 s; **(C)** Temperature distribution in can when *t* = 5,760 s; **(D)** Temperature distribution in can when *t* = 7,200 s;

As seen in Figure 2, the SHZ of the can does not appear in the geometric center of the can, which is due to the heat transfer from the outer wall to the SHZ in the can through solid–liquid transfer. Due to the existence of top clearance, the temperature in the can is uneven, forming non-isothermal flow. Considering the superposition effect of gravity, the slowest temperature rise position of the can is 19–25% from the bottom and 0–30% radius from the can center. At the beginning of the cooling stage, the temperature at the bottom and around the can rapidly decreases, and the cooling speed gradually slows down at the top and center. This may be due to the rapid condensation of water vapor at the top during the cooling process, the reduction of the flow rate of steam at the top of the can, and the slowing down of temperature transmissions. Compared with the heat transfer around and at the bottom of the can, the temperature at the top of the can drop more slowly.

#### 5.1.2. Prediction and analysis of the velocity field in the process of sterilization

**Figure. 3:**
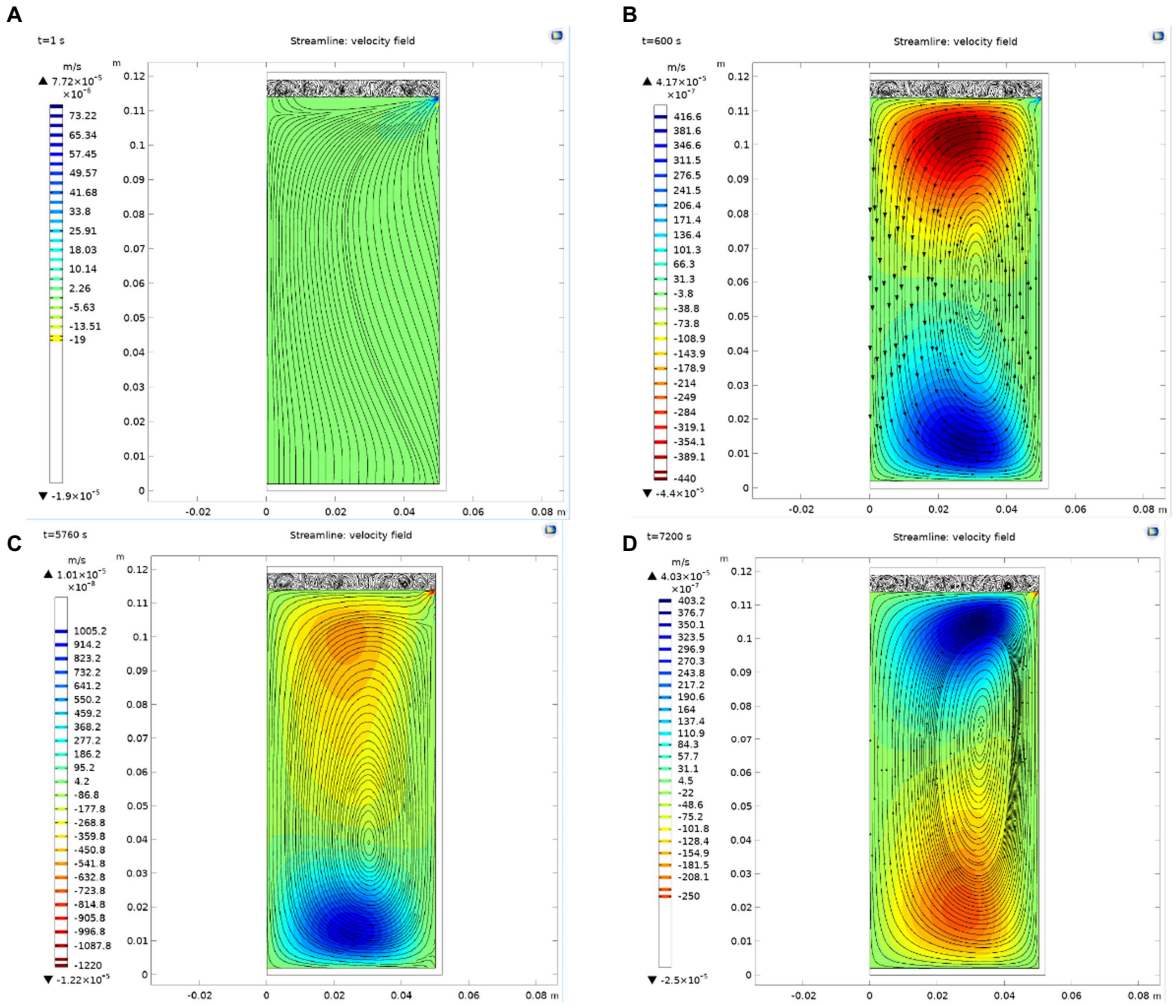
Variation in the velocity field in the can. **(A)** Flow distribution in can when *t* = 1 s; **(B)** Flow distribution in can when *t* = 600 s; **(C)** Flow distribution in can when *t* = 5,760 s; **(D)** Flow distribution in can when *t* = 7,200 s.

As shown in Figure 3, due to the temperature difference between the surface wall and the chestnut puree in the can, a material velocity flow from the surroundings to the center and from the bottom to the top is formed driven by the temperature difference. In the heating stage, due to the large temperature difference, the two strong velocity flows formed on the can wall and bottom tend to be dense with the reduction in the temperature difference, which indicates that the flow rate decreases. Moreover, this trend remains until the end of the constant temperature stage. In this process, the central point of the circulation is not unchanged but is slightly shifted with the change in the circulation. After cooling starts, the velocity flow quickly reverses the direction from the center of the can to the surrounding areas to form a circulation because the temperature outside the can decreases rapidly. From the perspective of flow lines, the flow lines are sparse at the beginning of cooling and then tend to be dense, indicating that the temperature difference gradually decreases at this time, and the cooling process is close to ending.

#### 5.1.3. Dynamic calculation of the *F* value and determination of the cold point position

**Figure. 4:**
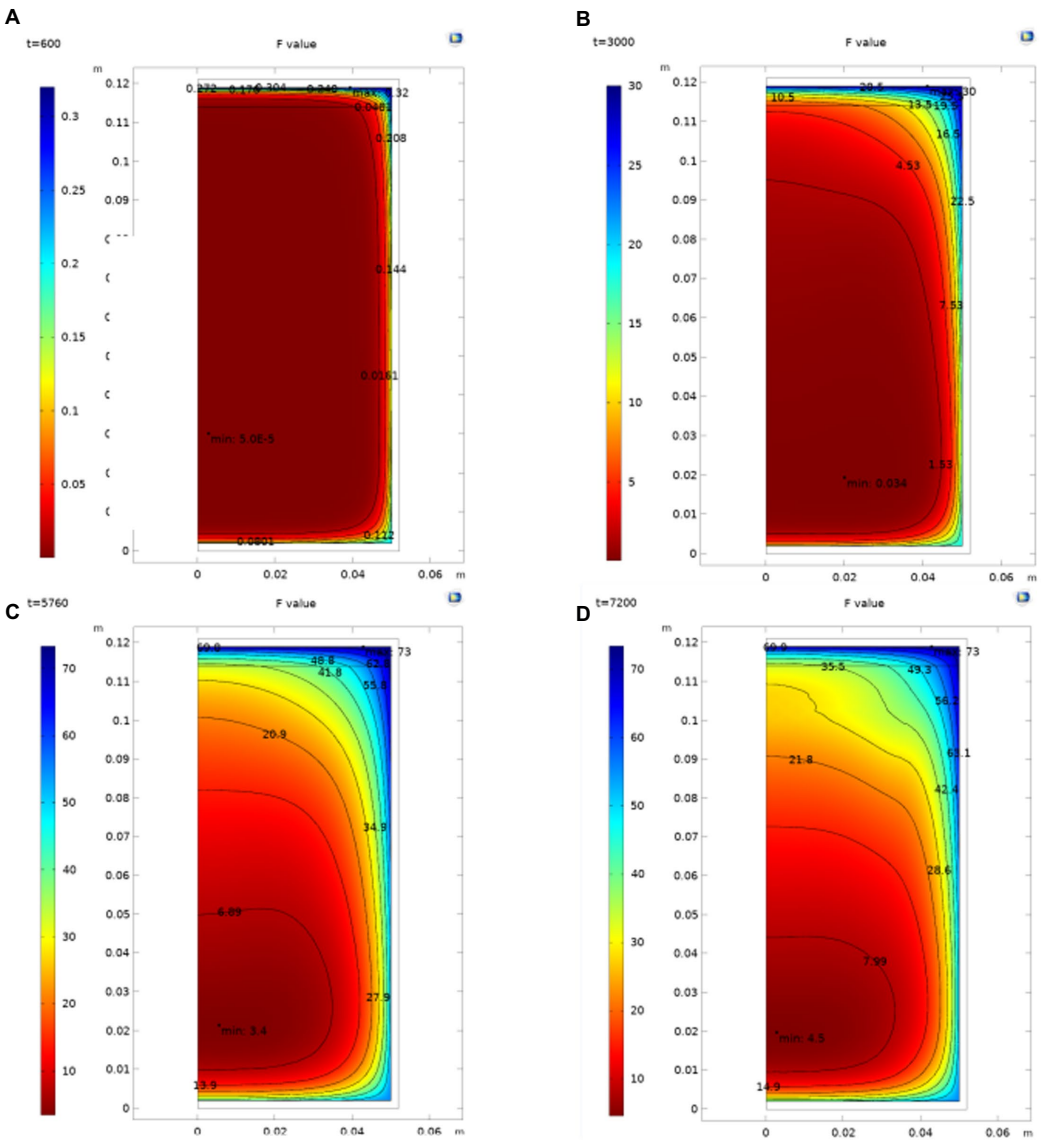
Distribution of the *F* value in the can. **(A)** the *F* value distribution in can when *t* = 600 s; **(B)** Flow distribution in can when *t* = 3,000 s; **(C)** Flow distribution in can when *t* = 5,760 s; **(D)** Flow distribution in can when *t* = 7,200 s.

Figure 4 shows that during the sterilization of cans, the point of the lowest instantaneous *F* value in the can is not fixed. In the heating stage, due to the large temperature change and rapid flow, the position of the lowest point of the cumulative sterilization *F* value of the cans changes greatly. With the entry of the constant temperature sterilization stage, the change of the lowest point of the cumulative sterilization *F* value is also gradually maintained after the temperature difference tends to be flat. Furthermore, in the cooling down stage, the temperature rapidly drops. It has little effect on the cumulative sterilization *F* value, so the position of the cumulative sterilization *F* value does not significantly change. The position of the SHZ in the can be obtained by determining the minimum value of F in the can after simulation. From the simulation in the figure, it can be seen that the cold point of chestnut puree is at the position *r* = 0.0027 m and *h* = 0.0188 m.

### 5.2. Test verification

**Figure. 5:**
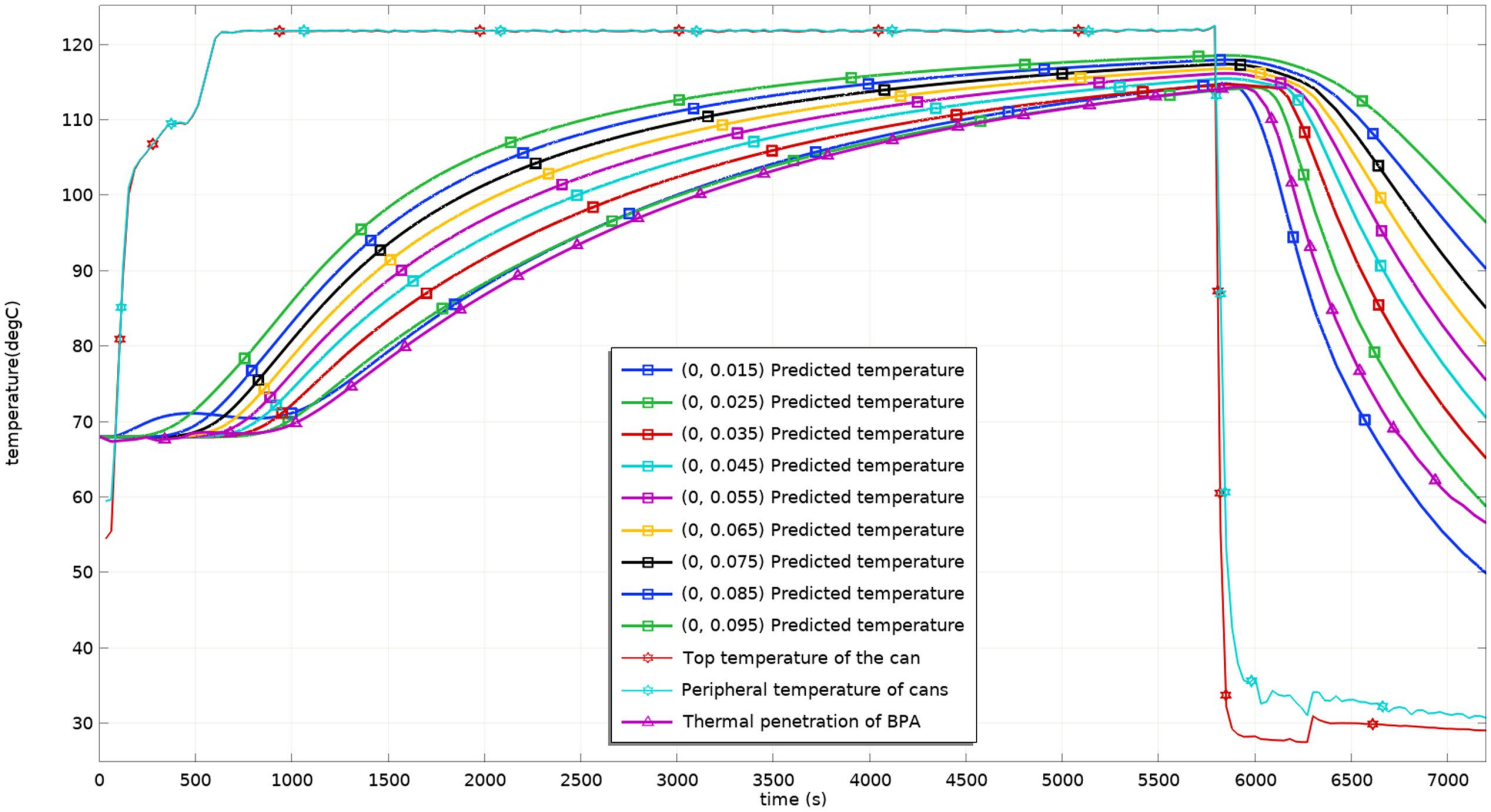
Predicted and tested values of temperature at different heights in the can before process adjustment.

Figure 5 shows the dynamic temperature values during the sterilization process of the virtual and actual models after the temperature probe is placed at the cold point. The curve of the model in the figure is basically consistent with the curve of the test measured value. The model better reflects the changing temperature process of the test, and the temperature field can be predicted with the help of the model. Considering factors such as the deviation of the probe from the material flow and the position of the probe in the test process, the model better reflects the situation of sterilization lethality, and the model can be used to adjust the process and predict the situation of the lethality field.

### 5.3. Process optimization

#### 5.3.1. Effect of the sterilization temperature on *F* value

**Figure. 6:**
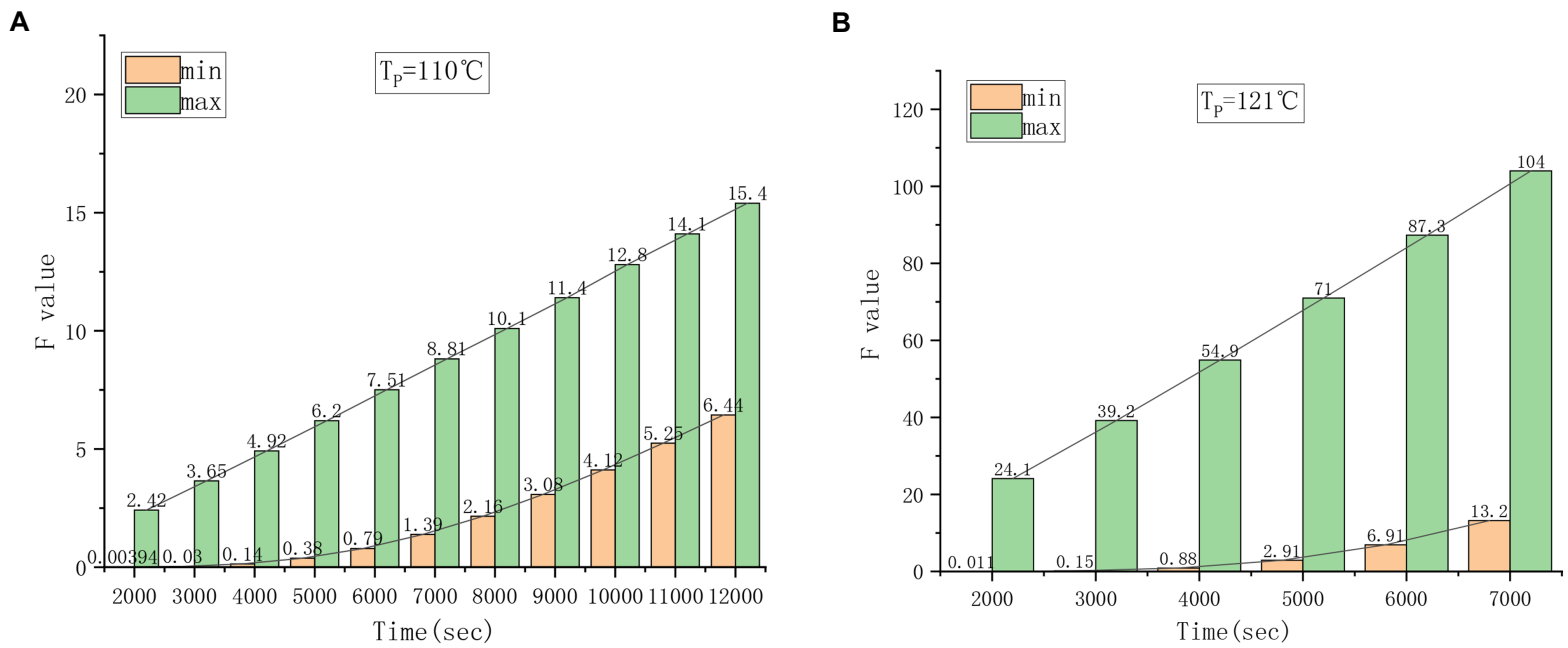
Changes in Fmax and Fmin at different sterilization temperatures. **(A)** 110°C sterilization temperature. **(B)** 121°C sterilization temperature.

Using the above heat transfer parameters, the sterilization temperature at 110 and 121°C is calculated through software. The results of the highest and lowest *F* values obtained by different sterilization times are shown in Figure 6. It can be seen from the comparison that the highest *F* value and the lowest *F* value are relatively close at 110°C (Figure 6A), while the highest *F* value and the lowest *F* value are significantly different at 121°C (Figure 6B). The change in the highest *F* value at different heating times is basically linear, and the change in the lowest *F* value has a nonlinear relationship with the sterilization temperature. On the premise of meeting the F0 value required by SHZ to achieve commercial sterility, it is suggested that a lower sterilization temperature is helpful to prevent an excessive difference between the maximum *F* value and the minimum *F* value in the can to achieve better quality products.

#### 5.3.2. Simulation and optimization

**Figure. 7:**
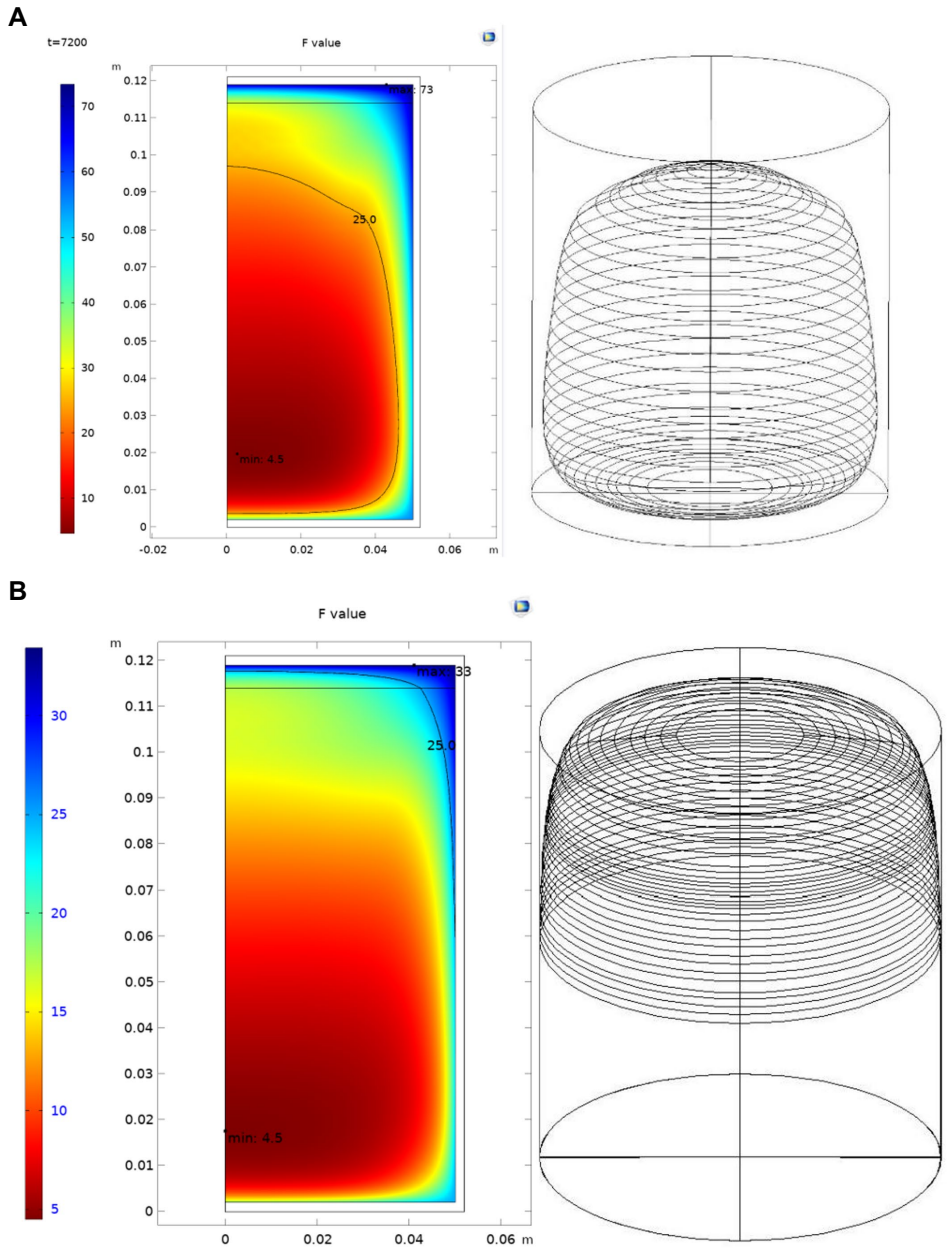
Areas of *F* value within 25 before and after process adjustments. **(A)** Before process adjustment. **(B)** After process adjustment.

Through computer simulation, the sterilization process was gradually adjusted. From the calculation results of the *F* value, it can be seen that the original sterilization formula is as follows: the initial temperature is 68–70°C, 10–86-24/121°C, the chestnut puree at the edge of the can is at a high temperature for a long time, and the accumulated *F* value is much higher than the *F* value at the cold point. This should be the reason for the massive browning of the surface wall of the can. From the analysis of the existing canned products, the browning part of the can wall is mostly within 5 ~ 10 mm of the surface wall. The *F* value of this part of the area is above 25, as shown in Figure 7. It can be estimated that the volume with an *F* value above 25 accounts for approximately 42.05% of the total volume.

On the one hand, from the perspective of the quality requirements, it is necessary to adjust the new sterilization process to improve the product quality and reduce the browning rate of the product. Under the condition of maintaining the initial temperature, appropriately reducing the maximum sterilization temperature of food and adopting the two-stage sterilization mode can help prevent the product quality deterioration caused by the excessive *F* value at the edge of the can wall due to the high temperature of the can wall. On the other hand, from the perspective of the production efficiency, a sterilization temperature that is too low causes the sterilization time of the products to be too long, which will affect the efficiency of enterprises. Therefore, it is necessary to balance the relationship between these two parameters.

Through computer simulations and adjustments, the two-stage sterilization method of 110°C and 118°C is adopted for simulation. The simulation results show that the sterilization type of 10–65–48-14/118–110°C is close to the original sterilization value and effectively avoids the problem of an excessive *F* value. Through the introduction of the CAD model, it can be estimated that an *F* value above 25 accounts for 5.99% of the total volume. This is more than 80% lower than that of the original process.

**Figure. 8:**
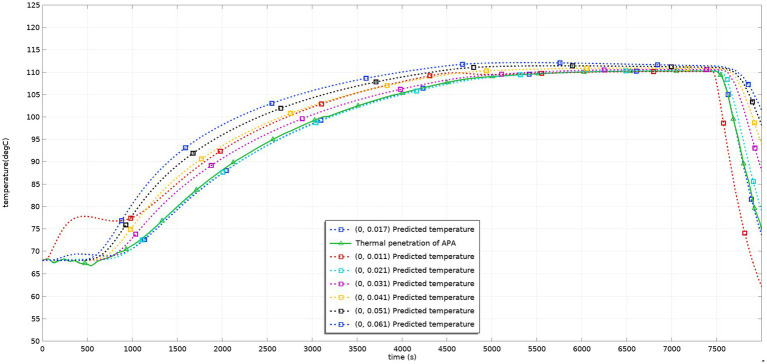
Predicted and tested values of the temperature at the SHZ after process adjustments.

The new adjusted process was used for sterilization. The temperature probe was placed at the lowest predicted *F* value (0, 0.018) again, and the measured data are shown in Figure 8. The results showed that the predicted temperature was close to the actual measured temperature during the whole sterilization process, and the total *F* value was 4.52. The predicted results predicted that the results more realistically reflected the actual situation.

#### 5.3.3. Product quality verification

**Figure. 9:**
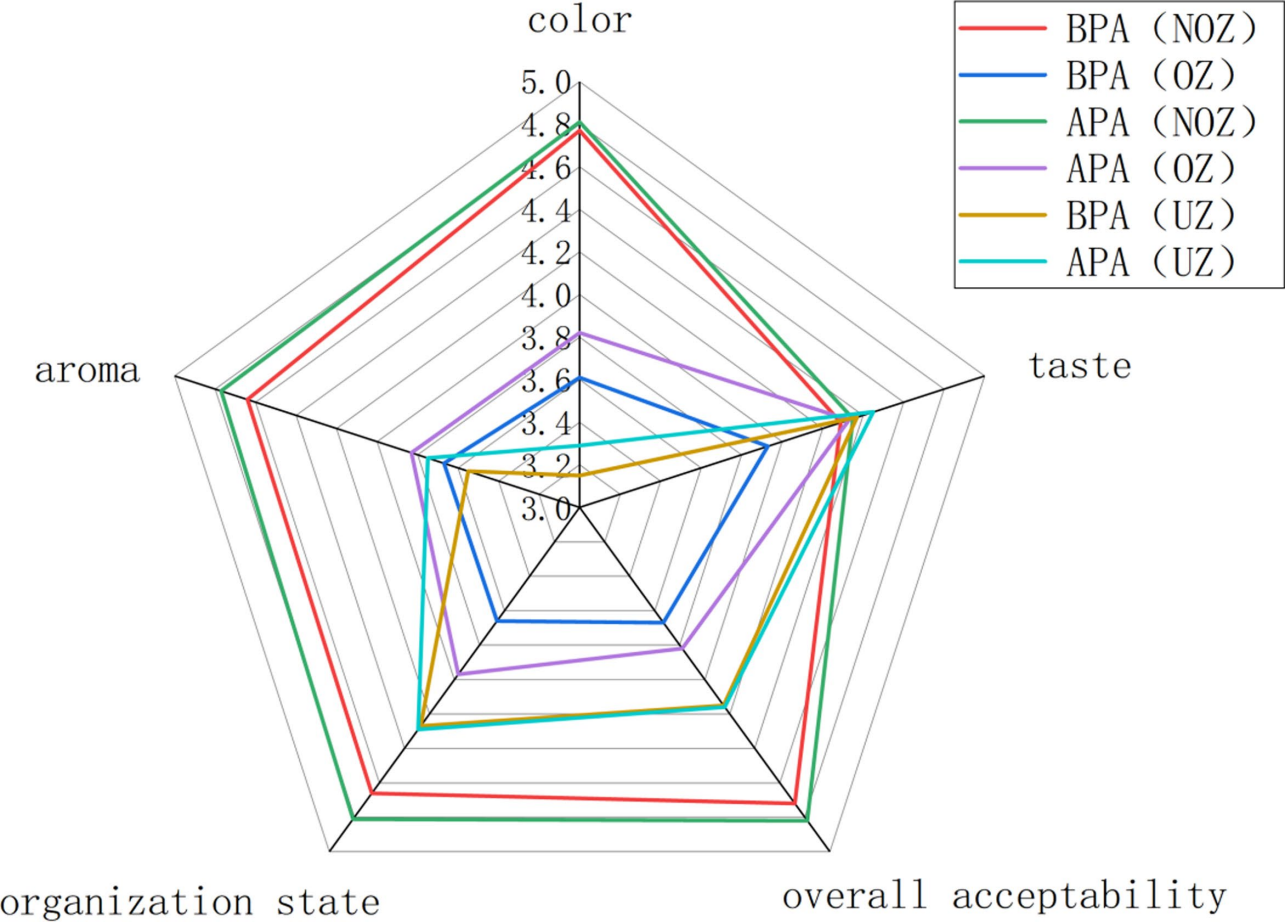
Sensory evaluation of products in different parts of cans.

Whether before or after the process adjustment, the products in the NOZ, OZ and top zone (TZ) were compared, and the results are shown in Figure 9. The sensory scores in terms of the color, shape, aroma and overall acceptability in the NOZ were significantly better than those in the OZ and the TZ. In addition, the sensory score in terms of the color in the TZ of the product was significantly lower than that in the NOZ and the OZ. This shows that the chestnut puree on the top of the can produce a certain degree of browning during the sterilization process, whether before or after adjustment, and the browning degree is deeper than that of the can wall. This may be because after the can is sealed, the residual oxygen in the can escapes to the top of the can with the increase in the heating temperature, which is related to the oxidation of chestnut puree on the surface.

## 6. Conclusion

Determining the canning sterilization process is difficult in the production of most canning enterprises. As mentioned above, there are many reasons for the browning of different parts of chestnut puree cans, which may be caused by excessive heating or oxidation reactions. In this study, the production process of chestnut puree products was numerically simulated using the finite element method. After the validity of the model was verified through experiments, the sterilization process was simulated, the results were predicted using the sterilization parameters of the modified model, and the new process was used to alleviate the browning problem of the can wall edges caused by excessive heating. The experiment shows that the numerical simulation can help optimize the sterilization process, reduce the number of experiments, and reduce manpower and material resources.

Outlook: In this test, we only focus on the external environment with uniform temperature and the internal environment with a single component. In the actual production process, there may be many situations, such as multi-can dense arrangements, multi-batch circulation of hot water recovery, rotary sterilization, and solid–liquid mixing. These heat transfer methods will be more complex and need to be further tested and studied in the future.

## Data availability statement

The original contributions presented in the study are included in the article/Supplementary material, further inquiries can be directed to the corresponding authors.

## Author contributions

WW conceptualized the experiments, draft preparation, analyzed the data, and designed the experiment. ZW performed the experiments. LH collected the experimental data. JZ supervised the experimentation. JH assisted with critical revision of the manuscript. YL analyzed the data. XP revised the manuscript. JW secured funding and revised the manuscript. All authors reviewed, revised, and approved the final manuscript.

## Funding

This work was financially supported by Fujian Provincial Science and Technology Department Spark Plan Project (project name: computer assisted improvement of Puree cans sterilization process and development and demonstration of refined products, number: 2021S0054) and Fujian Provincial Science and Technology Plan Project (project name: Zhangzhou Food IndustryTechnology Research Institute, number: 2018 N2002).

## Conflict of interest

JW is employed by Fujian Zishan Group Co., Ltd. Zhangzhou, China.

The remaining authors declare that the research was conducted in the absence of any commercial or financial relationships that could be construed as a potential conflict of interest.

This study received funding from Fujian Zishan Group Co., Ltd. Zhangzhou, China. The funder had the following involvement in the study: Simulation and optimization of the thermal sterilization process of puree cans using the production of chestnut puree as an example.

## Publisher’s note

All claims expressed in this article are solely those of the authors and do not necessarily represent those of their affiliated organizations, or those of the publisher, the editors and the reviewers. Any product that may be evaluated in this article, or claim that may be made by its manufacturer, is not guaranteed or endorsed by the publisher.
