# Supplementary material for: Simulation and optimization of the thermal sterilization process of puree cans using the production of chestnut puree as an example
Source: Front Microbiol. 2023 Apr 24;14:1135700. doi: 10.3389/fmicb.2023.1135700 (PMC10166203; doi:10.3389/fmicb.2023.1135700)
Supplement: Supplementary file 1 [file Data_Sheet_1.docx]

**Supplemental materials**

**Table S1 The density of chestnut mud measured at different temperatures**. All experimental datas were plotted as mean ± standard from triplicate determinations.

| temperature(K) | density(g/m^3^) |
| --- | --- |
| 303.15 | 1089.9±2.7 |
| 308.15 | 1087.7±2.8 |
| 313.15 | 1085.2±2.9 |
| 318.15 | 1083.8±3.1 |
| 323.15 | 1081.1±3.3 |
| 328.15 | 1078.7±3.4 |
| 333.15 | 1076.3±3.6 |
| 338.15 | 1074.3±3.8 |
| 343.15 | 1072.4±3.9 |
| 348.15 | 1069.8±4.2 |
| 353.15 | 1067.5±4.4 |
| 358.15 | 1065.3±4.5 |
| 363.15 | 1063.2±4.7 |
| 368.15 | 1061.8±4.9 |

**Table S2 The Viscosity of chestnut mud measured at different temperatures.** All experimental datas were plotted as mean ± standard from triplicate determinations.

| temperature(K) | Viscosity(Pa·s) |
| --- | --- |
| 293.15 | 95.8±4.6 |
| 303.15 | 69.3±3.9 |
| 313.15 | 53.7±3.3 |
| 323.15 | 44.4±2.9 |
| 333.15 | 39.1±2.6 |
| 343.15 | 35.8±2.2 |
| 353.15 | 33.9±2.1 |
| 363.15 | 32.7±2.1 |

**Table S3 Thermal conductivity of chestnut mud measured at different temperatures**. All experimental datas were plotted as mean ± standard from triplicate determinations.

| temperature(K) | Thermal conductivity(W/(m•K)) |
| --- | --- |
| 293.15 | 0.574012196±0.0059 |
| 303.15 | 0.573830549±0.0083 |
| 313.15 | 0.574555102±0.0097 |
| 323.15 | 0.576185855±0.012 |
| 333.15 | 0.578722808±0.017 |
| 343.15 | 0.582165961±0.020 |
| 353.15 | 0.586515314±0.023 |
| 363.15 | 0.591770867±0.026 |
